# Epigenetic Targets for Reversing Immune Defects Caused by Alcohol Exposure

**DOI:** 10.35946/arcr.v35.1.11

**Published:** 2013

**Authors:** Brenda J. Curtis, Anita Zahs, Elizabeth J. Kovacs

**Affiliations:** **Brenda J. Curtis, Ph.D.**, *is a postdoctoral fellow, in the Department of Surgery, director of research in the Burn & Shock Trauma Institute, and director of the Alcohol Research Program at Loyola University, Chicago, Illinois.*; **Anita Zahs, Ph.D.**, *is a postdoctoral fellow, in the Department of Surgery, director of research in the Burn & Shock Trauma Institute, and director of the Alcohol Research Program at Loyola University, Chicago, Illinois.*; **Elizabeth J. Kovacs, Ph.D.**, *is a professor and vice chair of research in the Department of Surgery, director of research in the Burn & Shock Trauma Institute, and director of the Alcohol Research Program at Loyola University, Chicago, Illinois.*

**Keywords:** Alcohol consumption, alcohol exposure, alcoholism, chronic drinking, binge drinking, epigenetics, epigenetic mechanisms, epigenetic targets, DNA code, immune system, immune cells, innate immunity, adaptive immunity, infections, inflammation, gut, respiratory system, acute respiratory syndrome (ARDS), liver cancer, alcoholic liver disease (ALD)

## Abstract

Alcohol consumption alters factors that modify gene expression without changing the DNA code (i.e., epigenetic modulators) in many organ systems, including the immune system. Alcohol enhances the risk for developing several serious medical conditions related to immune system dysfunction, including acute respiratory distress syndrome (ARDS), liver cancer, and alcoholic liver disease (ALD). Binge and chronic drinking also render patients more susceptible to many infectious pathogens and advance the progression of HIV infection by weakening both innate and adaptive immunity. Epigenetic mechanisms play a pivotal role in these processes. For example, alcohol-induced epigenetic variations alter the developmental pathways of several types of immune cells (e.g., granulocytes, macrophages, and T-lymphocytes) and through these and other mechanisms promote exaggerated inflammatory responses. In addition, epigenetic mechanisms may underlie alcohol’s ability to interfere with the barrier functions of the gut and respiratory systems, which also contribute to the heightened risk of infections. Better understanding of alcohol’s effects on these epigenetic processes may help researchers identify new targets for the development of novel medications to prevent or ameliorate alcohol’s detrimental effects on the immune system.

Extensive clinical and experimental data suggest that alcohol consumption has dose-dependent modulatory effects on the immune system that influence the two arms of the immune response (i.e., innate and adaptive immune responses). In many other organ systems, such as the brain and liver, alcohol consumption has been shown to alter factors that can modify gene expression without changing the DNA code (i.e., epigenetic modulators) and which play critical roles in mediating alcohol’s effects. However, very few studies have focused on the effects of alcohol-mediated epigenetic alterations on immunity. Because chronic alcohol consumption is correlated with an exacerbated state of chronic inflammation (which is part of the innate immune response), researchers can apply knowledge of how epigenetic factors are dysregulated in inflammatory and autoimmune disorders to identify potential epigenetic targets that can be used to develop therapies for treating alcohol-abusing patients. This review summarizes how inflammatory mediators and both innate and adaptive immune responses are modulated by moderate, binge, and chronic alcohol consumption. The discussion further identifies and highlights exciting potential avenues to explore epigenetic regulation of these immune responses.

## Epigenetics: An Overview

All cells within an organism carry identical genetic information in the form of DNA, yet a multitude of individual cell types arises during the course of development. These individualized cellular morphologies, characteristics, and functions result from the unique gene expression profiles of the different cell types. Regulation of gene expression profiles is critical not only during development, but also for cellular proliferation, differentiation, environmental adaptation, stress, and immune responses throughout the individual’s lifetime and is largely dependent on epigenetic mechanisms. An epigenetic trait is a stably heritable observable characteristic (i.e., phenotype) that results from changes in a chromosome without alterations in the DNA sequence (Berger et al. 2009). Epigenetic regulation can involve a variety of chemical modifications of the DNA (e.g., methylation) and the histone proteins around which the DNA is wrapped (e.g., methylation, acetylation, phosphorylation, ubiquitinylation, ADP-ribosylation, and sumoylation), as well as the actions of small molecules called noncoding microRNAs (miRNAs). Of these mechanisms, higher-than-normal DNA methylation (i.e., hypermethylation) and miRNAs generally are correlated with decreased protein production through gene-silencing mechanisms and posttranscriptional regulation (Carthew and Sontheimer 2009). Age, environment, and exposure to drugs and other toxins (e.g., alcohol) can directly influence the epigenetic profile of the organism (Feil and Fraga 2012).

It is well documented that alcohol exposure prior to an injury or infection dampens the immune system, resulting in a range of adverse outcomes, such as delaying infection clearance, extending hospital stays, and increasing morbidity and mortality compared with nonintoxicated patients (for a review, see Messingham et al. 2002). This has led to the development of the “two-hit hypothesis,” where the first hit (i.e., alcohol exposure) exaggerates the organism’s physiological responses to the second hit (i.e., injury or infection). Epigenetic memory may be a contributing factor in this process.

So how does the epigenetic memory work? Throughout evolution, eukaryotic cells have adapted so that a vast amount of genetic material has become organized and compacted into the nucleus by forming a higher-order structure known as chromatin. The basic building block of chromatin is the nucleosome, which comprises 147 base pairs of DNA wrapped around a core of eight small histone proteins. Nucleosomes undergo dynamic relaxation and condensation in the nucleus, a process requiring the activities of two sets of molecules:

- ATP-dependent chromatin remodeling complexes that physically tighten or loosen histone–DNA contacts; and
- Epigenetic modifying enzymes that add or remove posttranslational covalent modifications from the tails of the histone proteins, thus either allowing or preventing access of nuclear factors to the DNA that are needed for gene transcription.

Also known as the histone code, the intricate combination of covalent modifications on the histones directly influences DNA–histone binding by altering electrical charge and providing a specific docking signal for recruitment of chromatin-modifying complexes and transcriptional machinery to either block or promote active gene transcription (Jenuwein and Allis 2001; Strahl and Allis 2000). Some covalent modifications are typically associated with the same effect on transcription; for example, histone acetylation generally is associated with active gene transcription (Turner 2000). The effects of histone methylation are much more complex. Thus, the degree of methylation (i.e., mono-, di-, or trimethylation); the particular histone protein, and, more specifically, lysine residue(s) being modified (e.g., H3K4,^1^ H3K9, H3K27, H3K36, H3K79, or H4K20); and the degree of chromatin condensation (i.e., condensed heterochromatin versus relaxed euchromatin) all play a role. Likewise, the part of the gene where the DNA or histone modification occurs (i.e., the genomic location)—that is, whether it occurs in a promoter, enhancer, or the gene body—influences whether a gene will be actively transcribed after lysine methylation (Bannister and Kouzarides 2005; Heintzman et al. 2007; Martin and Zhang 2005).

Specific enzymes are responsible for adding or removing acetyl or methyl moieties from histone tails. Histone acetyl transferases (HATs) and histone deacetylases (HDACs) add and remove acetyl groups, respectively. Similarly, methylation is tightly regulated by enzymes that add methyl groups to (i.e., methyltransferases) or remove methyl groups from (i.e., demethylases) specific lysine residues (Shilatifard 2006). So far, 18 HDACs have been identified and subdivided into four classes. Classes I, II, and IV require Zn^2+^ for enzymatic activity, whereas class III HDACs, also known as sirtuins, utilize a mechanism that requires the cofactor nicotinamide adenine dinucleotide (NAD^+^) (Shakespear et al. 2011).

Several approaches may potentially be used to prevent or correct the epigenetic effects of alcohol consumption, such as alcohol-mediated immune defects. For example, inhibition of HDACs by molecular HDAC inhibitors (HDACis), alteration of DNA methylation on cytosine residues, or miRNA modulation all represent branches of possible therapeutic targets for restoring immune defects caused by alcohol exposure. These approaches will be discussed later in this review.

## Epigenetics and Alcohol

Beverage alcohol (i.e., ethanol) is predominantly metabolized by the enzymes alcohol dehydrogenase (ADH), cytochrome p450 (CYP 450), and aldehyde dehydrogenase (ALDH) in the liver (Dey and Cederbaum 2006). This process produces oxidative metabolites such as acetaldehyde, acetate, acetyl-CoA, and reactive oxygen species (ROS), as well as nonoxidative products, such as phosphatidylethanol (PEth) and fatty acid ethyl ester (FAEE) (Best and Laposata 2003; Shukla and Aroor 2006; Shukla et al. 2001). Many of these products or metabolites can induce tissue-specific epigenetic changes (Choudhury and Shukla 2008; Shukla and Aroor 2006). Ethanol exposure leads to epigenetic alterations through several mechanisms, including enhancing the enzymatic activity of HATs; altering substrate availability for histone acetylation, DNA, and histone methylation; or by influencing miRNA production. For example, studies found the following:

- Ethanol exposure enhances the activity of a HAT called p300 in the liver of rats fed a chronic ethanol diet, which leads to heightened histone acetylation (Bardag-Gorce et al. 2007).
- Elevated ROS levels resulting from ethanol metabolism increase histone H3 acetylation in liver cells (i.e., hepatocytes) (Choudhury et al. 2010).
- Chronic alcohol exposure can mediate a shift in the ratio of reduced NAD^+^ (NADH) to NAD^+^, and this reduced redox state suppresses the activity of the redox-sensitive HDAC, SIRT1, thus augmenting histone acetylation in rats (Bardag-Gorce and French 2007; You et al. 2008).
- Ethanol metabolism dramatically increases production of acetyl-CoA, which is used in histone acetylation by HATs; consequently, ethanol exposure and metabolism amplifies the amount of substrate available for histone acetylation (Yamashita et al. 2001).
- Ethanol exposure causes dysregulated methionine metabolism, resulting in diminished production of a molecule called S-adenosylmethionine (SAMe), which serves as a methyl-group donor for both DNA and histone methylation (Lu and Mato 2005; Mason and Choi 2005; Shukla and Aroor 2006).
- Chronic ethanol exposure decreases the levels of the antioxidant glutathione, which serves as the predominant scavenger of ROS in the liver (Choudhury and Park 2010; Lu et al. 2000); this glutathione reduction leads to both regionally and globally reduced DNA methylation (i.e., hypomethylation) (Lee et al. 2004; Lertratanangkoon et al. 1997).
- Chronic ethanol exposure in rats leads to inhibition of a set of reactions called the ubiquitin–proteasome pathway, which helps eliminate molecules that are defective or no longer needed from the cell. This inhibition of the ubiquitin–proteasome pathway likely alters protein turnover of transcription factors and histone-modifying enzymes and is associated with epigenetic alteration at a specific lysine residue (K9) of histone H3 (i.e. increased H3K9-ac and reduced H3K9-me2) as well as DNA hypomethylation (Oliva et al. 2009).
- Acetylation of H3K9 also is associated with increased ADH1 expression in cultured rat hepatocytes treated with 100 mM ethanol for 24 hours, suggesting that ethanol (and its metabolites) may amplify ethanol metabolism (Park et al. 2005).

Through the various mechanisms discussed above, alcohol consumption can lead to multifactorial, dose-dependent, and tissue-specific epigenetic effects. For example, cultured primary rat hepatocytes demonstrated a dose- and time-dependent histone-acetylation response to ethanol exposure. Thus, cells treated with 5–100 mM ethanol for 24 hours exhibited a maximal, eightfold increase in H3K9-ac levels at 24 hours following treatment with the highest ethanol concentration (Park et al. 2003). Furthermore, histone acetylation seemed to be selective for the H3K9 residue, because acetylation of other H3 lysines (i.e., K14, K18, and K23) was unaffected by ethanol exposure (Park and Lim 2005; Park and Miller 2003). Similar findings were obtained in hepatic stellate cells (Kim and Shukla 2005). Finally, another study (Pal-Bhadra et al. 2007) found that hepatocytes treated for 24 hours with 50 mM and 100 mM ethanol also exhibited altered histone methylation status, resulting in increased H3K4 dimethylation (H3K4-me2) and decreased H3K9 dimethylation (H3K9-me2). However, unlike histone lysine acetylation, which was restored to baseline levels 24 hours after ethanol withdrawal in cultured hepatocytes, changes in histone lysine methylation status were not reversed and may provide a long-term epigenetic memory (Pal-Bhadra et al. 2007).

Ethanol metabolites, including acetaldehyde and acetate, also could cause H3K9-specific acetylation in rat hepatocytes. Interestingly, the signaling pathways activated by acetate and ethanol seemed to modulate H3K9-ac via different mechanisms. Thus, certain molecules (i.e., inhibitors of enzymes known as mitogen-activated protein kinases) prevented acetylation by ethanol but had no effect on the acetate-dependent formation of H3K9-ac (Park and Lim 2005). In addition to acetylation, ethanol and acetaldehyde exposure also promotes phosphorylation of histone H3 at serines 10 and 28 (Lee and Shukla 2007). Whereas ethanol exposure lead to similar phosphorylation levels at both serine 10 and serine 28, acetaldehyde generated greater phosphorylation at serine 28 than at serine 10 (Lee and Shukla 2007). These studies indicate that the complexity of ethanol-induced epigenetic changes increases even further when taking into account that ethanol metabolites also trigger epigenetic effects that may differ from those produced by ethanol exposure.

Rat models of acute/binge and chronic alcohol exposure have been utilized to examine the relationship between epigenetic gene regulation and alcohol exposure in vivo. In one of those models, a single dose of ethanol diluted in sterile water resulting in a concentration of 6 grams ethanol per kilogram bodyweight (g/kg) was injected directly into the stomachs of 8-week-old male Sprague-Dawley rats. This high-dose binge-alcohol exposure model was used to compare H3K9 modification status across 11 different tissues at 1, 3, and 12 hours following ethanol exposure (Kim and Shukla 2006). The investigators found that in the testes, this alcohol exposure caused robust global increases in H3K9-ac at 1 hour but not at later time points. In contrast, in the lung and spleen robust increases in H3K9-ac were apparent at all three time points. In the liver, no marked elevation in H3K9-ac was observed at early (i.e., 1- or 3-hour) time points, but a profound elevation occurred at 12 hours. In addition, in the blood vessels, pancreas, colorectum, stomach, heart, brain, and kidney, no change in H3K9-ac was observed at any time-point tested. Finally, methylation of H3K9 was not altered in any tissue (Kim and Shukla 2006).

Other investigators evaluated changes in gene expression levels after chronic ethanol treatment using in vivo models. One of these models is the Tsukamoto-French rat model of alcoholic liver disease (Tsukamoto et al. 1985), in which male Wistar rats were fed a liquid diet containing a constant amount of alcohol (13 g/kg/day) for 30 days using an intragastric feeding tube. This treatment, which resulted in a 6- to 10-day cyclic pattern of urinary alcohol level (UAL) peaks (about 500 mg%) and troughs (about 100 mg%) (Bardag-Gorce and French 2002), allowed the investigators to compare gene expression profiles at high and low blood alcohol levels (BALs) by microarray analyses. These analyses identified dramatic changes in gene expression levels in the livers of the alcohol-treated rats. Overall, approximately 1,300 genes were dysregulated between BAL cycles (French et al. 2005), prompting additional studies aimed at elucidating the epigenetic contribution of alcohol-mediated transcriptional dysregulation in the liver and other tissues (Bardag-Gorce and French 2007; Kim and Shukla 2006; Park and Lim 2005). Furthermore, UAL peaks were associated with increased levels of the HAT, p300, which specifically transfers acetyl groups to H3K9 residues. This finding at least partially explains the selective H3K9 acetylation observed both in vitro and in vivo in correlation with ethanol exposure (Bardag-Gorce and French 2007). Finally, studies assessing the effects of changes in epigenetic mechanisms resulting from inhibition of the ubiquitin–proteasome pathway (using a drug called PS-341) or from chronic ethanol exposure in rats using the Tsukamoto-French model found increases in H3K9-ac levels, decreases in H3K9-me2 levels, and increased p300 levels in liver nuclear extracts (Oliva and Dedes 2009). These findings suggest that chronic ethanol exposure alters transcriptional regulation of a plethora of genes through many mechanisms that affect epigenetic modulators.

In summary, both acute/binge and chronic alcohol exposure can result in tissue- and cell-specific patterns of epigenetic responses. Future studies to determine the precise role of alcohol-mediated chromatin modifications hopefully will identify new epigenetic targets and pathophysiological mechanisms for regulating gene expression in diseases associated with alcohol consumption. The factors contributing to altered epigenetic modifications arising from acute versus chronic alcohol exposure may differ, because chronic alcohol exposure has been strongly correlated with nutrient deficiencies and a shift in the redox state. This implies that potential therapeutic interventions targeting epigenetic modifiers may need to differ depending on the degree of alcohol consumption. Furthermore, understanding the role of nutrients in regulating epigenetic modifications will provide insight into potential dietary supplementation in chronic alcohol-abusing patients.

## Alcohol and the Immune System

**Figure 1:**
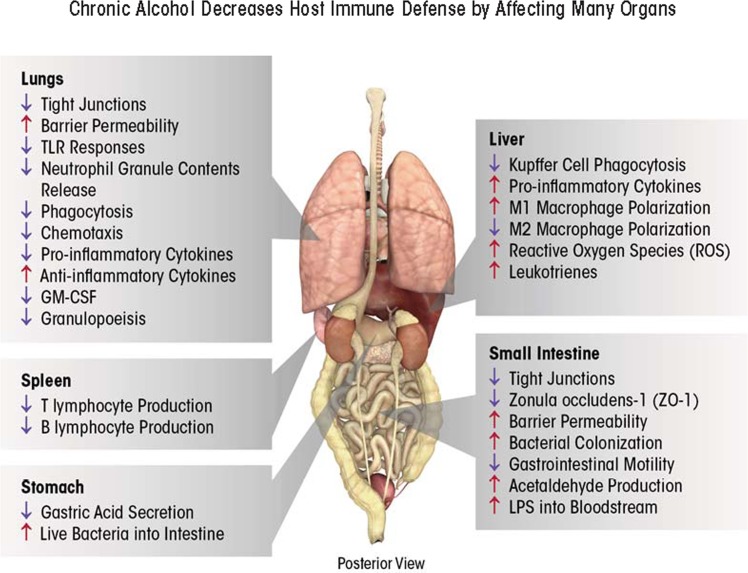
Chronic alcohol exposure causes immune dysfunction through effects on multiple organs. In the lungs, excessive inflammation causes tissue damage, increasing barrier permeability, and dampening many cellular immune responses, such as recognizing bacteria (through toll-like receptors [TLRs]), attacking pathogens (through phagocytosis), decreasing production of granulocytes (i.e., granulocytopenia) as well as their migration (i.e., chemotaxis), and altering important signaling and recruiting molecules (e.g., GM-CSF and chemokines). In the spleen, alcohol consumption affects immunity by decreasing T- and B-lymphocyte production. In the stomach, alcohol decreases gastric acid levels, allowing live bacteria to pass into the small intestine. Combined with decreased gastrointestinal motility, a byproduct of alcohol metabolism (i.e., acetaldehyde) increases intestinal barrier permeability by weakening cell–cell junctions, and allows bacterial toxins (i.e., lipopolysaccharide [LPS]) to pass into the bloodstream. LPS damages the liver, leading to excessive release of pro-inflammatory cytokines, leukotrienes, and ROS into the circulation. In addition, alcohol in the liver can alter macrophage (Kupffer cell) polarization and decrease phagocytosis.

A recent report from the Centers for Disease Control and Prevention (CDC) stated that alcohol abuse in the form of binge drinking (defined by the CDC as four or more drinks for women and five or more drinks for men in a short period of time) is the third-leading preventable cause of death in the United States, resulting in more than 80,000 deaths each year and enormous economic costs (i.e., more than $220 billion in 2006) (CDC 2012). A significant, positive correlation exists between the duration and amount of alcohol consumed and the risk for developing several serious medical conditions, including acute respiratory distress syndrome (ARDS) (Boe et al. 2009; Moss et al. 1999); liver cancer (i.e., hepatocellular carcinoma) (McKillop and Schrum 2009; Yamauchi et al. 1993); and alcoholic liver disease (ALD), which encompasses cirrhosis, hepatitis, and fibrosis (Gramenzi et al. 2006; Mann et al. 2003). Binge and chronic consumption (defined as more than eight drinks per day) renders patients more susceptible to various types of infection, such as hepatitis C virus infection in the liver and opportunistic infections in the respiratory system (e.g., ARDS and pneumonia), and advances the progression of HIV infection, largely through dysregulated immune responses (Baliunas et al. 2010; Bhatty et al. 2011; Prakash et al. 2002; Romeo et al. 2007*b*; Zhang et al. 2008) (figure 1).

The mammalian immune system is an elaborate network of molecules and cells that identify, combat, and eliminate harmful agents; it can be divided into two branches: innate and adaptive immunity. The innate immunity is present from birth, whereas the adaptive immunity develops over the organism’s life course with the continuous exposure to pathogens and other potentially harmful compounds.

### The Innate Immune Response

Following pathogen or toxin exposure, the ancient innate immune response is responsible for immediate recognition, rapid attack, and destruction of foreign intruders and involves inflammatory reactions. Innate immune cells carry special molecules called Toll-like receptors (TLRs) on their surface that recognize and bind highly conserved structures on bacterial, fungal, or viral surfaces, including peptidoglycan, flagellin, zymosan, and lipopolysaccharide (LPS, also known as endotoxin) (Janeway and Medzhitov 2002). The innate-immune cells also activate the adaptive immune response by digesting the foreign intruders and then presenting certain molecules derived from these pathogens (i.e., antigens) on their surface for recognition by adaptive immune cells. This antigen presentation, which initiates the adaptive immune response and provides a “memory” of the initial recognition of the antigen, allows for a rapid immune response if the same infection occurs again in the future.

An important subset of innate immune cells are macrophages; they eliminate pathogens by a process called phagocytosis^2^ and then present pathogen-derived molecules on their surface to activate adaptive immune cells. Macrophages can have alternate names based on their anatomical location; for example, macrophages residing in the liver are called Kupffer cells. Furthermore, macrophages can be subdivided into two groups based on their functional phenotype (Martinez et al. 2008) (see table 1):

- Classically activated (M1) macrophages, whose activation results in a proinflammatory response.
- Alternatively activated (M2) macrophages, whose activation results in an anti-inflammatory response.

**Figure 2:**
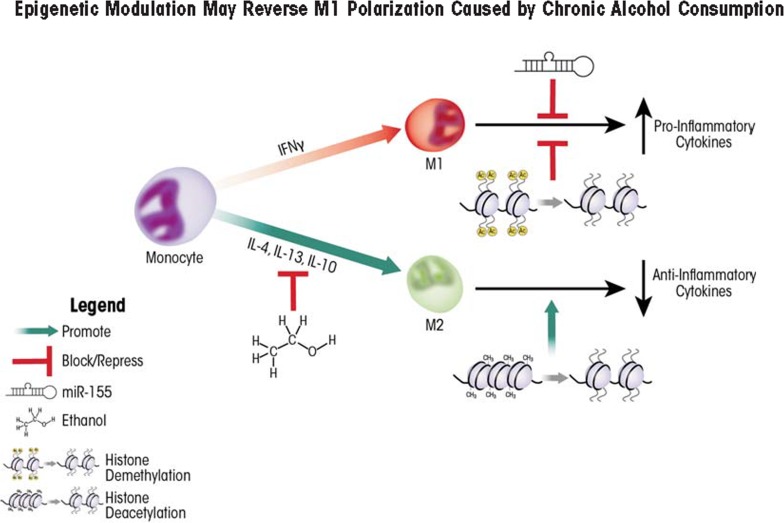
Chronic alcohol consumption skews macrophage polarization toward an M1 (i.e., pro-inflammatory) phenotype, leading to excessive or prolonged inflammation. Two approaches using epigenetic modulators—microRNA 155 (miR-155) and histone deacetylase inhibitors—can potentionally reverse protein translation or gene transcription of M1 pro-inflammatory cytokines. Another type of enzyme—histone lysine (H3K27) demethylases—increase transcription of M2 anti-inflammatory cytokines. Factors that increase protein levels or enhance activity of H3K27 demethylases therefore may potentially be utilized to promote M2 polarization.

After challenge to the immune system occurs (e.g., an infection), macrophages are generated by the maturation of precursor cells called monocytes. During this process, the macrophages can become either M1 or M2 macrophages; this is called macrophage polarization. The ratio of M1 to M2 macrophages changes depending on the presence of a variety of factors; this variability is known as macrophage plasticity and allows the organism to modulate the immune response. Accordingly, controlling macrophage plasticity is critical to first battle pathogens and then resolve the resulting inflammation to prevent tissue damage. Alcohol exposure skews macrophage polarization towards M1 (i.e., towards inflammation) in the liver (Louvet et al. 2011; Mandal et al. 2011), resulting in deleterious consequences (figure 2).

Dendritic cells (DCs) are an additional component of the innate immune response. They have an important role in linking the innate and adaptive branches of the immune system. To this end, the DCs exhibit proteins called major histocompatibility complex (MHCs) on their surface. With the MHC proteins, DCs present antigens to other cells that are part of the adaptive immune system—that is, B and T lymphocytes (also known as B and T-cells). DCs mature following stimulation by whole bacteria or LPS or after exposure to various signaling molecules, such as interleukin 1β (IL-1β), granulocyte macrophage colony–stimulating factor (GM-CSF), and tumor necrosis factor-α (TNFα) (Winzler et al. 1997). The mature DCs migrate to lymphoid organs to prime and activate naïve T-cells (Lee and Iwasaki 2007). Activated T-cells then complete the immune response by producing and releasing specific signaling molecules (i.e., cytokines) that will stimulate other innate immune cells or interact with B-cells, leading to the development of immune molecules (i.e., antibodies). Mature DCs also secrete high levels of IL-12 (Reis e Sousa et al. 1997), enhancing both innate and adaptive immune responses (summarized in table 2).

Alcohol consumption has a variety of effects on innate immune cells. For example, alcohol decreases the phagocytic activity of monocytes, macrophages, Kupffer cells, microglia, and DCs and diminishes their capacity to present antigens and produce the molecules necessary for microbe killing. In addition, alcohol alters expression of other proteins (i.e., pathogen pattern recognition receptors) on their cell surface that are required for cell–cell interactions among immune cells (for reviews, see (Goral et al. 2008; Karavitis and Kovacs 2011; Romeo and Warnberg 2007*b*). Furthermore, the levels of a type of immune cell called granulocytes often are very low in alcoholics with severe bacterial infections, which has been strongly correlated with increased mortality (Perlino and Rimland 1985). Finally, rodent models have demonstrated that following infection, alcohol significantly decreased both phagocytic activity and production of the signaling molecule granulocyte colony-stimulating factor (G-CSF) in a TNFα-dependent manner (Bagby et al. 1998) as well as blocked differentiation or maturation of granulocytes (i.e., granulopoiesis) (Zhang et al. 2009).

### The Adaptive Immune Response

B-cells, T-cells, and antigen-presenting cells (APCs) are key players of the adaptive immune response. Like DCs, APCs present antigen to B and T-cells that have not yet been activated (i.e., naïve B and T-cells), contributing to their maturation and differentiation. Naïve T-cells are classified based on expression of specific proteins on their surface called cluster of differentiation (CD) proteins. Two of those proteins important in distinguishing different T-cell populations are CD4 and CD8. T-cells carrying the CD8 protein (i.e., CD8^+^ cells) ultimately gain the ability to recognize and kill pathogens (i.e., become cytolytic T-cells). Conversely, CD4^+^ T-cells give rise to several T helper (Th) cell subsets, including Th1, Th2, and Th17 cells, that will produce mutually exclusive groups of cytokines which help mount specific immune responses by stimulating other immune cells (Zygmunt and Veldhoen 2011) (table 3). Alcohol exposure can promote the development of Th2 cells over the other helper-cell populations. This shift in T helper differentiation towards Th2 is correlated strongly with defective immune responses as well as increased rates of infection, morbidity, and mortality (Cook et al. 2004; Romeo and Warnberg 2007*b*).

## The Effects of Alcohol Exposure on Innate Immune Cells and the Potential Role of Epigenetics

### Epigenetics Play a Crucial Role in Innate Immune-Cell Differentiation and Maturation

During the early stages of blood cell formation (i.e., hematopoiesis), the developing cells fall into one of two developmental paths: the myeloid lineage, which includes granulocytes and monocytes (which then further differentiate into macrophages or DCs), and the lymphoid lineage, which includes B- and T-lymphocytes. This myeloid versus lymphoid lineage commitment corresponds with global and reduced DNA methylation, respectively (Ji et al. 2010). During infection, alcohol suppresses the development and maturation of granulocytes (i.e., granulopoiesis) (Zhang et al. 2009). Factors that increase DNA methylation, and therefore promote myeloid cell commitment, may serve as potential therapeutic targets for increasing granulocyte populations. Similarly, epigenetic factors play a crucial role in regulating monocyte terminal differentiation into DCs. Proper functioning of monocyte cells requires the expression of CD14, because it recognizes and binds LPS. DCs, however, do not utilize CD14, but instead require CD209 (DC-SIGN). Therefore, when monocytes differentiate into DCs, they lose expression of *CD14*, which is correlated with loss of epigenetic modifications associated with active transcription, including H3K9-Ac and H3K4me3. Concurrently, epigenetic changes occur within the *CD209* locus, leading to increased *CD209* transcription. The increase in *CD209* transcription is associated with loss of epigenetic modifications typically associated with transcriptional silencing, including DNA methylation and formation of H3K9me3 and H3K20me3 (Bullwinkel et al. 2011). In the future, therapeutics that specifically target epigenetic modifications within the *CD14* or *CD209* loci may be designed to direct monocyte terminal differentiation towards one particular cellular fate (Bullwinkel et al. 2011).

### Epigenetic Regulation of Macrophage Polarization

Alcohol alters macrophage polarization in the liver—that is, it alters the normal ratio of M1 to M2 macrophages. Chronic alcohol exposure sensitizes Kupffer cells to LPS stimulation, leading to prolonged and predominant M1 polarization and the exacerbated release of pro-inflammatory cytokines (Mandrekar and Szabo 2009; Thakur et al. 2007). This shift in macrophage polarization is reversible, because recent studies demonstrated that a hormone produced by adipose cells (i.e., adiponectin), can shift Kupffer cells isolated from chronic alcohol-exposed rat livers towards M2 polarization (Mandal and Pratt 2011).

Another potential strategy for shifting Kupffer cell polarization is the use of therapeutic reagents that target epigenetic modifiers because epigenetic processes play central roles in the regulation of immune-system functions. For example, one critical mechanism to restore the internal balance (i.e., homeostasis) of the immune system in response to infection involves miRNA-dependent post-transcriptional regulation. Researchers found that expression of one specific miRNA called miR-155 was dramatically increased when macro -phages derived from the bone marrow were stimulated by LPS. This enhanced miRNA expression served to fine-tune the expression of pro-inflammatory mediators and promote M2 polarization (Ruggiero et al. 2009). Similarly, ethanol exposure also can affect miR-155 expression. When a specific macrophage cell line (i.e., the RAW 264.7 macrophage cell line) was treated with 50 mM ethanol (corresponding to a BAL of 0.2 g/dl, which commonly is observed in chronic alcoholics), miR-155 expression was significantly enhanced (Bala et al. 2011). Ethanol treatment prior to stimulation with LPS further augmented miR-155 production, and a linear, significant correlation existed with increased TNFα production, likely because miR-155 increased TNFα mRNA stability (Bala and Marcos 2011). Finally, a murine model of ALD confirmed increased miR-155 and TNFα levels in Kupffer cells isolated from ethanol-treated animals compared with control animals, suggesting that miR-155 is an important regulator of TNFα in vivo and likely contributes to the elevated TNFα levels often observed in chronic alcoholics (Bala and Marcos 2011).

Besides ethanol-induced production of miR-155, histone modifications also can regulate macrophage polarization. As mentioned earlier, macrophages and other innate immune cells carry TLRs on their surface that can interact with LPS and other molecules, leading to the activation of the TLRs. Studies have demonstrated that when TLR4 was stimulated by LPS, histone acetylation and H3K4 tri-methylation (both of which are associated with active gene transcription) occurred in DNA regions encoding several pro-inflammatory cytokines (Foster et al. 2007; Takeuch and Akira 2011). Macrophage stimulation using the cytokine IL-4 and LPS also induced expression of an H3K27 histone lysine demethylase enzyme called Jumonji Domain Containing-3 (JmjD3/Kdm6b), causing transcription of specific M2-associated genes (De Santa et al. 2007; Satoh et al. 2010). The role of this demethylase is further supported by studies using cultured cells or mice in which specific genes were inactivated (i.e., knockout mice) that demonstrated that JmjD3/Kdm6b activity was not required for mounting antibacterial M1 responses, but was essential for M2 responses following exposure to a molecule (i.e., chitin) found in fungi and other parasites (Bowman and Free 2006; Satoh and Takeuchi 2010). Taken together, these findings suggest that epigenetic regulation of factors that specifically alter macrophage polarization may be able to shift and/or restore the normal M1/M2 physiological balance in alcohol-exposed patients (also see table 1 and figure 2).

## The Effects of Alcohol Exposure on Adaptive Immunity and the Potential Role of Epigenetics

### The Potential Role of Epigenetics in Reversing Th2 Polarization

Alcohol exposure impairs IL-12 production by DCs and IL-23 production by macrophages, thereby skewing T helper cell commitment towards a Th2 lineage (Happel et al. 2006; Mandrekar et al. 2004). Lysine methylation at histone H3K27 plays an important role in regulating transcription of the *IL-12* gene and thereby regulating DC activation (Wen et al. 2008). Accordingly, the development and use of drugs that target H3K27-specific histone methyltransferases or demethylases to treat diseases associated with alcoholism are a promising, future endeavor (see table 2).

**Figure 3:**
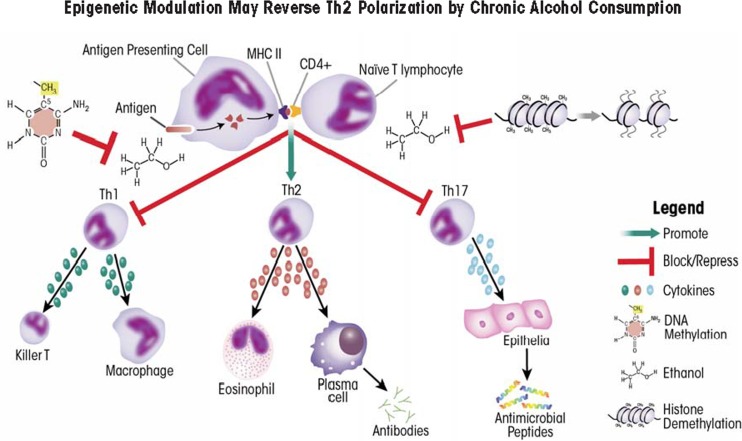
Alcohol-induced T helper cell polarization towards a Th2 phenotype suppresses immune responses. Alcohol decreases IL-12 production by antigen presenting cells, resulting in fewer naïve T-cell differentiating into Th1 cells, and blocks the release of IL-23 from macrophages, thereby preventing Th17 differentiation. Methylation of DNA or histones (H3K27) may reverse Th2 polarization.

T-cell production also is modulated by alcohol consumption, but at least some of the effects may be both gender- and dose-dependent. For example, moderate daily consumption of one beer by women or two beers by men for 30 days caused significantly higher abundance of CD3^+^ T-cells in women, but not in men (Romeo et al. 2007*a*). Conversely, in male mice, chronic alcohol exposure was correlated with decreased CD4^+^ and CD8^+^ T-cells in the spleen and thymus (Saad and Jerrells 1991) and increased free (i.e., soluble) CD8 in the blood. This soluble CD8 can bind T-cell receptors, block activation by APCs, and thus impede viral clearance (Jerrells et al. 2002), indicating a way through which chronic alcoholism can impair the immune response. These findings indicate that drugs that can enhance cytokine production by the limited, inefficient T-cells found in alcoholics may restore the immune response. HDACis may be one such approach because histone deacetylation inhibits transcription of the gene encoding IL-4 (i.e., *Il4*) and inhibition of deacetylation accordingly could promote IL-4 production (Valapour et al. 2002). Drugs targeting DNA methylation also may be beneficial because DNA methylation plays an important role in regulating the transition of naïve T-cells to either Th1 or Th2 cell fates. Specifically, when naïve T-cells transition into Th2 cells, certain regions of the Il4 loci (specifically the 5′ region) become hypomethylated. Conversely, when transitioning to Th1 cells, the 3′ region of *Il4* becomes hypermethylated, demonstrating that a highly complex system of methylation/demethylation mediates T helper cell differentiation (Lee et al. 2002; Mullen et al. 2002). Treatment of T-cell lines with an agent called 5-azacytidine, which inhibits DNA methylation, leads to the production of cytokines not normally produced by these cells, including IL-2 and IFNγ (Ballas 1984; Young et al. 1994). This effect may help to restore the defective Th1 response in patients abusing alcohol (also see table 3 and figure 3).

## The Effects of Chronic Alcohol and Inflammation and the Potential Role for Epigenetics

Chronic alcoholism is correlated with excessive or prolonged inflammation, caused in part through an overactive innate immune response and elevated oxidative stress (Khoruts et al. 1991). Studies have demonstrated that circulating levels of the pro-inflammatory cytokines TNFα, IL-1β, and IL-6 were much higher in alcoholics than in healthy nondrinkers (Khoruts and Stahnke 1991). The higher circulating levels of these cytokines resulted from increased production of pro-inflammatory cytokines by circulating monocytes and resident tissue macrophages, including Kupffer cells (for a review, see Cook 1998). These cells were also more sensitive to stimulation by LPS, which further exacerbated TNFα secretion and contributed to cytotoxicity (Schafer et al. 1995). The increased sensitivity to LPS stimulation partially was caused by decreased production of the anti-inflammatory cytokine, IL-10, which negatively regulates TNFα secretion by monocytes (Le Moine et al. 1995). Thus, chronic alcohol exposure disrupts the delicate and precise regulation of inflammatory regulators.

To assess alcohol’s effects on the inflammatory responses of macrophages, researchers have used a human monoblastic cell line, MonoMac6, which has many features of mature macrophages and has been used to model Kupffer cell responses (Zhang et al. 2001). Preliminary studies demonstrated that prolonged (i.e., 7 day) exposure of these cells to high-dose (86 mM) ethanol dramatically enhanced pro-inflammatory cytokine responses following LPS stimulation and was correlated with increased histone H3 and H4 global acetylation, as well as elevated acetylation of specific cytokine gene promoters, including those encoding *IL-6* and *TNF* (Kendrick et al. 2010). This increased acetylation was dependent upon conversion of ethanol to its metabolites, acetate and acetyl-coA, by two enzymes called acetyl-coenzyme A synthetase short-chain family members 1 and 2 (ACSS1 and ACSS2) and also was associated with a significant decrease in HDAC activity (Kendrick and O’Boyle 2010). Interestingly, unlike with rat hepatocytes and hepatic stellate cells, no global modulation of histone acetylation was observed with acute ethanol treatment (Kendrick and O’Boyle 2010).

ACSS1 and ACSS2 only are activated for acetate and acetyl-CoA formation during ethanol metabolism but not during normal sugar metabolism that also results in acetyl-CoA generation. Therefore, they represent an exciting potential therapeutic target for reducing the exacerbated inflammatory response observed with chronic alcohol exposure because their depletion should not alter normal cellular metabolism and energy generation. Another potential approach to restoring cytokine homeostasis may be to reduce proinflammatory cytokine transcription by administering drugs that increase HDAC recruitment to actively transcribed chromatin (e.g., theophylline), thereby counteracting the decreased HDAC activity induced by chronic ethanol exposure (Kendrick and O’Boyle 2010).

Although drugs that modulate epigenetic targets have not yet been used specifically to treat alcohol-induced inflammation, research of other inflammatory and autoimmune diseases suggest that epigenetic modulation plays a critical role in regulating the inflammatory cytokine network (Ballestar 2011; Halili et al. 2009; Rodriguez-Cortez et al. 2011). Accordingly, agents that normalize this epigenetic modulation (e.g., HDACis) are a promising therapy for the treatment of inflammatory and autoimmune diseases, including the exacerbated inflammation observed with chronic alcohol exposure. HDACis are efficacious in animal models of inflammatory bowel disease, septic shock, graft-versus-host disease, and rheumatoid arthritis (Bodar et al. 2011; Halili and Andrews 2009; Joosten et al. 2011; Reddy et al. 2004, 2008). Furthermore, the HDACi vorinostat has been used in clinical trials for reducing the severity of graft-versus-host disease in patients with bone marrow transplants (Choi and Reddy 2011), and the HDACi givinostat has been studied for the treatment of several other inflammatory conditions. These HDACis originally were developed to increase transcription of genes that induce cell death (i.e., apoptosis) of malignant cells. The doses of HDACi required to diminish inflammatory processes, however, are dramatically lower than the doses required for cancer treatment, and minimal side effects have been reported (Dinarello 2010; Vojinovic and Damjanov 2011). The importance of lysine acetylation as a regulatory mechanism has been supported by a study characterizing the entirety of all proteins that are acetylated in the human body (i.e., the human lysine acetylome). This study identified 1,750 proteins that could be acetylated on lysine side chains, including proteins involved in diverse biological processes, such as the processing of mRNAs (i.e., splicing), cell-cycle regulation, chromatin remodeling, and nuclear transport (Choudhary et al. 2009). In fact, protein acetylation may be as important as phosphorylation in governing cellular processes (Choudhary and Kumar 2009; Kouzarides 2000). For example, acetylation of proteins in the fluid filling the cell (i.e., the cytosol) can either activate or block essential signaling cascades and may partially explain how low-dose HDACi treatment decreases the production of pro-inflammatory cytokines (Dinarello et al. 2011).

It is important to note that the development of selective HDACis may be complicated by the fact that most HDACs are components of multi-protein complexes, which often include other HDACs (Downes et al. 2000; Fischle et al. 2001). Therefore, it is possible that inhibition of one HDAC inadvertently may alter the activity of other HDACs present in the complex. It also is likely that some functional redundancy exists among HDACs as well as within the biological inflammatory pathways they regulate. Moreover, the role of individual HDACs is tissue and cell-type specific; accordingly, development of specific HDACi molecules for treatment of each particular inflammatory disease will require cell- or tissue-targeting components.

## Alcohol Abuse and Leaky Barriers

Another important component of the innate immune system are the epithelial cells that line the outer surfaces of exposed tissues, such as the skin, respiratory, gastrointestinal (GI), and urogenital tracts. These cells provide a physical barrier that impedes pathogen invasion by forming strong intercellular associations (Tam et al. 2011; Turner 2009). Another critical function of epithelial cells in the innate immune system is their production of cytokines and chemokines in response to pathogen detection. (Elias 2007; Izcue et al. 2009; Parker and Prince 2011; Quayle 2002; Schleimer et al. 2007; Tracey 2002). Alcohol abuse is strongly correlated with defective, leaky barriers, particularly in the GI and respiratory tracts (Bhatty and Pruett 2011; Purohit et al. 2008).

### The Effect of Alcohol on the Gut and the Potential Role of Epigenetics

Chronic alcohol consumption increases microbial colonization and LPS accumulation in the small intestine by decreasing gastric acid secretion in the stomach and delaying GI motility (Bienia et al. 2002; Bode and Bode 1997; Bode et al. 1984). The intestinal epithelial barrier must allow water and nutrients to pass freely, yet prevent transfer of larger macromolecules. Whereas the epithelial cells themselves are impermeable to substances dissolved in water (i.e., hydrophilic solutes), the space between the cells (i.e., paracellular space) must be sealed to maintain this barrier function. A leaky intestinal barrier is deleterious because it allows transfer of potentially harmful macromolecules and bacterial products (e.g., LPS) into the blood and lymph (Rao 2009). If it reaches the liver, LPS can target multiple cell types there, including Kupffer cells, neutrophils, hepatocytes, sinusoidal endothelial cells, and stellate cells (Brun et al. 2005; Duryee et al. 2004; Hoek and Pastorino 2002; Paik et al. 2003). Activation of these cells results in the release of pro-inflammatory mediators, such as ROS, leukotrienes, chemokines, and cytokines (e.g., TNFα and IL-1β), thereby directly contributing to liver damage and prolonged inflammation in chronic alcohol-abusing patients (Albano 2008; Brun and Castagliuolo 2005; Khoruts and Stahnke 1991; McClain et al. 2004).

The multifactorial contributions of chronic alcohol consumption to the development of ALD largely have been deciphered using rodent models. For example, investigators demonstrated a direct translocation of LPS across the gut mucosa in rats continuously administered alcohol directly into the stomach for 9 weeks (Mathurin et al. 2000). Other studies using mice in which the TNF-receptor 1 (TNF-R1) was removed (i.e., TNF-R1 knockout mice) and that were treated continuously with alcohol for 4 weeks determined that the alcohol-induced presence of LPS in the blood (i.e., endotoxemia) led to the release of TNFα from Kupffer cells, that in turn played a direct role in ALD (Yin et al. 1999). TNFα production is negatively regulated by H3K9 methylation (Gazzar et al. 2007), indicating that histone methylation can play a role in regulating inflammatory processes. This observation suggests that the prolonged inflammatory state associated with chronic alcohol exposure partially may be controlled by drugs targeting H3K9-specific demethylase enzymes.

Although alcohol itself does not alter intestinal permeability, one of the products of alcohol metabolism (i.e., acetaldehyde) increases barrier permeability in a dose-dependent manner (Basuroy et al. 2005) by disrupting intercellular connections, including both tight and adherens junctions (Atkinson and Rao 2001). One of the critical proteins ensuring the functionality of tight junctions is called zonula occludens 1 (ZO-1), and disrupted ZO-1 complexes are strongly correlated with increased intestinal barrier permeability (Walker and Porvaznik 1978). Interestingly, studies using a human intestinal cell line called Caco-2 found that ZO-1 production is regulated by microRNA-212 (miR-212). When these cells were cultured in the presence of 1 percent alcohol for 3 hours, they contained 71 percent less ZO-1 compared with cells not treated with alcohol. Moreover, the expression of miR-212 increased with alcohol treatment in a concentration-dependent manner; thus, cells treated with 1 percent alcohol for 3 hours had 2-fold higher expression of miR-212. These changes corresponded with defective tight junction morphology. Importantly, studies of colon samples taken from patients with ALD found significantly increased miR-212 expression compared with healthy control subject, and this increase paralleled a decrease in ZO-1. These findings demonstrate that miR-212 may play an important role in leaky intestinal barriers in ALD patients (Tang et al. 2008).

### The Effect of Alcohol on the Respiratory System and the Potential Role of Epigenetics

Mucosal organ leakiness also contributes to respiratory infections, partially by altering tight junctions between epithelial cells lining the air sacs in lungs where gas exchange occurs (i.e., the alveoli) (Simet et al. 2012). This leaky barrier provides the ideal opportunity for bacteria normally found in the body (i.e., commensal bacteria), such as *Streptococcus pneumoniae*, to invade the tissues and become pathogenic (Bhatty and Pruett 2011). In fact, alcohol consumption is correlated with increased incidence of community-acquired pneumonia, with approximately 50 percent of adult pneumonia patients reporting a history of alcohol abuse (Goss et al. 2003). Furthermore, alcohol abuse worsens complications from pneumonia (Saitz et al. 1997) and increases mortality (Harboe et al. 2009) in a dose-dependent manner (Samokhvalov et al. 2010). Alcohol also shifts the cytokine balance in the lung, contributing to the development of ARDS (Boe and Vandivier 2009; Crews et al. 2006; Moss and Steinberg 1999).

When an infection occurs, neutrophils and monocytes are recruited to the lungs (Goto et al. 2004). Upon activation, monocytes differentiate into alveolar macrophages, which play a crucial role in the clearance of *S. pneumoniae* (Goto and Hogg 2004). Rodent models have demonstrated that chronic alcohol exposure contributed to increased infection susceptibility by causing mucosal organ leakiness, as well as defective leukocyte recruitment and decreased neutrophil maturation, adhesion, chemotaxis, and phagocytosis. These changes partly resulted from faulty production of important signaling molecules, including G-CSF, GM-CSF, IL-8, IL-6, macrophage inflammatory protein (MIP-2), and CXC chemokine cytokine-induced neutrophil chemoattractant (CINC) (Boe et al. 2001). Alcohol also affected anti-inflammatory mediators by increasing the production of IL-10 and TGF-β (Boe and Vandivier 2009). Furthermore, chronic alcohol exposure inhibited the responses of CD8^+^ T-cells, which increased the morbidity and mortality associated with influenza virus infection (Meyerholz et al. 2008), and decreased IFNγ production following infection with *Klebsiella pneumoniae* (Zisman et al. 1998) in murine models.

Several strategies targeting epigenetic regulatory mechanisms may be effective in the treatment of alcohol-induced lung infections. For example, therapies that restore neutrophil recruitment to infected lungs through regulation of cytokine production would be beneficial. In support of this notion, it was demonstrated that pretreatment with G-CSF prior to alcohol exposure and *K. pneumoniae* infection was protective in mouse models (Nelson et al. 1991). Targeting miRNAs for treatment of inflammatory lung diseases, such as ARDS, offers an additional, novel therapeutic approach because the production of several miRNAs, including miR-9, miR-146a, miR-147, miR-148, and miR-152, was induced by LPS stimulation in mouse lungs (Bazzoni et al. 2009; Liu et al. 2009, 2010; Nahid et al. 2009; Taganov et al. 2006; Tili et al. 2007; Zhou et al. 2011). Several of these upregulated miRNAs created a negative feedback loop to prevent excessive production of pro-inflammatory cytokines, therefore contributing to immune regulation and homeostasis (Bazzoni et al. 2009; Liu et al. 2009, 2010). Although most research focused on understanding the role of miRNAs in inflammatory lung disease has been performed using animal models, future studies using human cell lines, tissues, and eventually patient samples clearly are warranted.

## Summary

The relationship between alcohol exposure and altered immune responses is complex. Chronic alcohol abuse is correlated with increased susceptibility to infection and causes tissue damage from an overactive innate immune response, excessive oxidative stress, and exacerbated or prolonged inflammation. Alcohol exposure has tissue- and immune cell-type–specific effects, such as influencing cell recruitment to infected or inflamed tissue, altering cytokine and chemokine production and secretion, skewing differentiation towards a particular cell fate or preventing cell replication, impairing antigen presentation, interfering with phagocytosis and granulopoiesis, or inducing apoptosis. Although the specific role of epigenetic modulation in this alcohol-induced immune dysregulation has not yet been determined, research in related fields strongly suggests that experimental and clinical studies are warranted.

## Figures and Tables

**Table 1:** Macrophages, Alcohol, and Potential Epigenetic Targets

	**Subtype**	**Factors Contributing to Activation^1^**	**Major Roles Following Activation^1^**	**Defects Caused by Chronic Alcohol^2^**	**Potential Epigenetic Targets**
Macrophages	M1 (Classical)	IFNγ Microbes	Engulf necrotic cells, toxic substances, and pathogens ↑ pro-inflammatory cytokines and reactive oxygen species (ROS) for direct pathogen killing and recruitment of other immune cells	Leads to predominant M1 polarization^3^ Kupffer cells sensitized to endotoxin stimulation^3,4^ ↑ Pro-inflammatory cytokines ↓ Phagocytic activity^5^ ↓ Capacity to present antigen^6^	miR-155 promotes M2 polarization^7^ Histone lysine demethylase, JmjD3, promotes transcription of M2-specific genes^8,9^
	M2 (Alternative)	Parasites Cytokines released by Th2, NK, basophils	↑anti-inflammatory cytokines Promote angiogenesis Promote wound healing	Macrophage polarization skewed towards M1 phenotype^3^	

SOURCES:

1 Gordon and Taylor, 2005,

2 Goral et al., 2008,

3 Thakur et al., 2007,

4 Mandrekar and Szabo, 2009,

5 Karavitis and Kovacs, 2011,

6 Szabo et al., 1993,

7 Ruggiero et al., 2009,

8 De Santa et al., 2007,

9 Satoh et al., 2010.

**Table 2:** Dendritic Cells, Alcohol, and Potential Epigenetic Targets

**Factors Contributing to Activation^1^**	**Major Roles Following Activation^2^**	**Defects Caused by Chronic Alcohol**	**Potential Epigenetic Targets**
Whole bacteria LPS IL-1β GM-CSF, TNFα	Migrate to lymphoid organs and present antigens to naïve T and B lymphocytes ↑IL-12 to enhance innate and adaptive immunity^5^	↓ IL-12 production^3^	Histone lysine methylation (H3K27) controls transcription of the IL-12 gene^4^

SOURCES:

1 Winzler et al., 1997,

2 Lee and Iwasaki, 2007,

3 Reis e Sousa et al, 1997,

4 Mandrekar et al., 2004,

5 Wen et al., 2008.

**Table 3:** T-Cells, Alcohol, and Potential Epigenetic Targets

**T-Cells Subtype**	**Major Roles Following Activation by Specific Antigen-Presenting–Cell Interaction**	**Defects Caused by Chronic Alcohol**	**Potential Epigenetic Targets**
CD8+Cytolytic T-cells	Direct pathogen killing	↓ CD8 + production in spleen and thymus^1^ ↑ soluble CD8→ blocks APC activation^2^	
CD4+T helper 1 (Th1)	↑ IFNγ → activates macrophages and cytolytic T-cells	↓ CD4 + production in spleen and thymus^1^ ↓IL-12 production by DC → ↓Th1 lineage specification^3^	↓ DNA methylation → ↑transcription of the gene coding for IFNγ (*Ifng*)^4^
CD4+T helper 2 (Th2)	↑ IL-4, IL-5, IL-13 → activates eosinophils ↑antibody production by plasma cells Important for humoral immunity and allergic response	↓ CD4^+^ production in spleen and thymus^1^ ↓Th1^+^ and ↓Th17→ Th2 predominates	↑ DNA methylation → ↓ transcription of gene coding for IL-4 (Il4)^5^ ↑ histone acetylation → ↓ Il4 transcription^6^
CD4+T helper 17 (Th17)	↑ IL-17, IL-17F, IL-21, IL-22, IL-23, IL-26 → ↑Antimicrobial peptides Important for mucosal barrier maintenance and immunity	↓ CD4+ production in spleen and thymus^1^ ↓IL-23 production by macrophages → ↓Th17 lineage specification^7^	

SOURCES:

1 Saad and Jerrells, 1991,

2 Jerrells et al., 2002,

3 Mandrekar et al., 2004,

4 Young et al., 1994,

5 Lee et al., 2002,

6 Valapouret al., 2002,

7 Happel et al., 2006.

## Footnotes

1 The standard nomenclature for histone modifications is to indicate both the histone protein (e.g., histone 3) and the specific amino acid affected. For example, in H3K4, the fourth lysine (abbreviated as K) of histone 3 is affected by epigenetic modification.

2 During phagocytosis, the macrophage engulfs the foreign pathogen, thus ingesting it into the cell, where it is degraded and eliminated.
